# Abemaciclib as initial therapy for advanced breast cancer: MONARCH 3 updated results in prognostic subgroups

**DOI:** 10.1038/s41523-021-00289-7

**Published:** 2021-06-22

**Authors:** Stephen Johnston, Joyce O’Shaughnessy, Miguel Martin, Jens Huober, Masakazu Toi, Joohyuk Sohn, Valérie A. M. André, Holly R. Martin, Molly C. Hardebeck, Matthew P. Goetz

**Affiliations:** 1 Breast Unit, The Royal Marsden NHS Foundation Trust, London, UK; 2 Baylor University Medical Center, Texas Oncology, US Oncology, Dallas, TX USA; 3 Medical Oncology Service, Hospital General Universitario Gregorio Marañón, Universidad Complutense, Madrid, Spain; 4 University of Ulm, Ulm, Germany; 5 Breast Cancer Unit, Kyoto University Hospital, Kyoto University, Kyoto, Japan; 6 Yonsei Cancer Center, Seoul, South Korea; 7 Eli Lilly and Company, Paris, France; 8 Eli Lilly and Company, Indianapolis, IN USA; 9 Department of Oncology, Mayo Clinic, Rochester, MN USA

**Keywords:** Breast cancer, Metastasis, Phase III trials, Breast cancer

## Abstract

In MONARCH 3, continuous dosing of abemaciclib with an aromatase inhibitor (AI) conferred significant clinical benefit to postmenopausal women with HR+, HER2− advanced breast cancer. We report data for clinically prognostic subgroups: liver metastases, progesterone receptor status, tumor grade, bone-only disease, ECOG performance status, and treatment-free interval (TFI) from an additional 12-month follow-up (after final progression-free survival [PFS] readout). In the intent-to-treat population, after median follow-up of approximately 39 months, the updated PFS was 28.2 versus 14.8 months (hazard ratio [HR], 0.525; 95% confidence interval, 0.415–0.665) in abemaciclib versus placebo arms, respectively. Time to chemotherapy (HR, 0.513), time to second disease progression (HR, 0.637), and duration of response (HR, 0.466) were also statistically significantly prolonged with the addition of abemaciclib to AI. Treatment benefit was observed across all subgroups, as evidenced by objective response rate change from the addition of abemaciclib to AI, with the largest effects observed in patients with liver metastases, progesterone receptor-negative tumors, high-grade tumors, or TFI < 36 months. Extended follow-up in the MONARCH 3 trial further confirmed that the addition of abemaciclib to AI conferred significant treatment benefit to all subgroups, including those with poorer prognosis.

The development and approval of cyclin-dependent kinase 4 and 6 (CDK4 & 6) inhibitors for hormone receptor-positive (HR+), human epidermal growth factor receptor 2-negative (HER2−) advanced breast cancer (ABC) has changed the treatment paradigm of this disease by significantly extending progression-free survival (PFS) and overall survival (OS)^1–4^. Abemaciclib is the only CDK4 & 6 inhibitor currently approved to treat HR+, HER2− ABC on a continuous dosing schedule as monotherapy (in the USA)^5^, or in combination with fulvestrant^6^ or a nonsteroidal aromatase inhibitor (AI)^7^.

Previously, an exploratory subgroup analysis of over 1000 patients from the final PFS readout of two phase 3 studies, MONARCH 2 and MONARCH 3, identified clinico-pathological factors with significant prognostic value. These included liver metastases, progesterone receptor (PgR) status, tumor grade, bone-only disease, Eastern Cooperative Oncology Group (ECOG) performance status, and treatment-free interval (TFI)^8^. Poorer prognosis was observed in patients with liver metastases, PgR-negative tumors, high-grade tumors, or shorter TFI (<36 months). All subgroups benefited from the addition of abemaciclib in terms of PFS and objective response rate (ORR) including those with poorer prognosis^8,9^.

More recently, a statistically significant OS benefit of abemaciclib in combination with fulvestrant was established in the MONARCH 2 trial (median OS benefit of 9.4 months), in patients with HR+, HER2− ABC who had progressed on prior endocrine therapy (ET)^3^. The OS benefit was consistent across subgroups, including patients with poorer prognosis. Exploratory efficacy endpoints such as time to first subsequent chemotherapy (TCT) and time to second disease progression or death (PFS2) may assist in understanding the impact of abemaciclib post-discontinuation, particularly since the OS data are still maturing in the MONARCH 3 trial.

To provide further insights on the longer-term benefit of the addition of abemaciclib in the intent-to-treat (ITT) population and in previously identified statistically significant prognostic subgroups, we report an additional 12-months follow-up of updated PFS and tumor response, as well as TCT and PFS2, which are important intermediate endpoints in the absence of mature OS data.

Between November 18, 2014 and November 11, 2015, 493 patients were randomized to receive abemaciclib plus AI (*n* = 328) or placebo plus AI (*n* = 165) in the MONARCH 3 trial^7^. At the October 31, 2018 cutoff, 88 (26.8%) patients in the abemaciclib arm and 20 (12.1%) patients in the placebo arm remained on treatment. The median follow-up was 39.0 months and 39.6 months in the abemaciclib and placebo arms, respectively.

PFS events occurred in 170 (51.8%) and 123 (74.5%) patients in the abemaciclib and placebo arms, respectively. Consistent with the primary analyses, the updated PFS was significantly improved with the addition of abemaciclib to an AI (stratified hazard ratio [HR], 0.525; 95% confidence interval [CI], 0.415–0.665; *p* < .0001; Fig. 1a). Median PFS was 28.2 months in the abemaciclib arm and 14.8 months in the placebo arm. The 3-year PFS rate was 41.3% in the abemaciclib arm versus 16.1% in the placebo arm. Consistent benefit from the addition of abemaciclib to AI was observed across all subgroups, including those with a poorer prognosis. Among subgroups, the largest treatment benefit was observed in patients with high-grade tumor (HR, 0.322; 95% CI, 0.190–0.546), TFI < 36 months (HR, 0.418; 95% CI, 0.240–0.729), PgR status negative (HR, 0.427; 95% CI, 0.265–0.687), or presence of liver metastasis (HR, 0.449; 95% CI, 0.259–0.777; Fig. 1b).

**Fig. 1:**
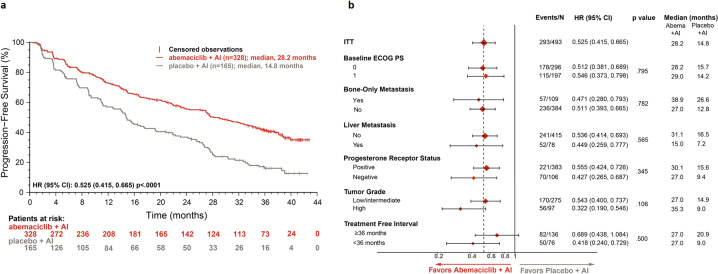
Progression-free survival in the intent-to-treat population and prognostic subgroups in MONARCH 3. **a** Kaplan–Maier analysis of investigator-assessed updated progression-free survival in the intent-to-treat population. **b** Forest plot of progression-free survival across intent-to-treat population and clinically prognostic subgroups. Abbreviations: Abema, abemaciclib; AI, aromatase inhibitor; CI, confidence interval; ECOG PS, Eastern Cooperative Oncology Group performance status; HR, hazard ratio; ITT, intent-to-treat.

In the ITT population, 301 patients received a post-discontinuation therapy (PDT, *n* = 178, 54.3% in the abemaciclib arm vs *n* = 123, 74.5% in the placebo arm). At any time post-discontinuation, 175 patients (*n* = 93, 28.4% in the abemaciclib arm vs *n* = 82, 49.7% in the placebo arm) received chemotherapy (Supplementary Fig. 1). ET was received by 246 patients (*n* = 145, 44.2% in the abemaciclib arm vs *n* = 101, 61.2% in the placebo arm). Overall, the most used treatments anytime post-discontinuation were fulvestrant, exemestane, capecitabine, and paclitaxel.

At the time of updated cut-off, a total of 380 (77.1%) patients had discontinued study treatment, 238 (72.6%) in the abemaciclib arm and 142 (86.1%) in the placebo arm. Overall, the distribution of patients receiving PDT in both arms was in line with expectations given the proportion of patients still on treatment in each arm. The addition of abemaciclib delayed initiation of first chemotherapy (stratified HR, 0.513; 95% CI, 0.380–0.691). In the abemaciclib arm, 47 (14.3%) died prior to receiving any chemotherapy compared to 13 (7.9%) patients in the placebo arm.

Most patients received ET as their first line of subsequent therapy (*n* = 132, 40.2% in the abemaciclib arm vs *n* = 88, 53.3% in the placebo arm), followed by chemotherapy (*n* = 39, 11.9% in the abemaciclib arm vs *n* = 30, 18.2% in the placebo arm), targeted therapy (*n* = 35, 10.7% in the abemaciclib arm vs *n* = 32, 19.4% in the placebo arm), and other therapies (*n* = 13, 4.0% in the abemaciclib arm vs *n* = 13, 7.9% in the placebo arm). Fulvestrant (15.5% in the abemaciclib arm vs 27.9% in the placebo arm) and letrozole (11.0% in the abemaciclib arm vs 8.5% in the placebo arm) were the most common agents administered after progression.

PFS2 was also statistically significantly prolonged with the addition of abemaciclib to AI. Median PFS2 across the ITT population was 36.9 months versus 25.6 months in the abemaciclib versus placebo arms (stratified HR, 0.637; 95% CI, 0.495–0.819). Consistent with the ITT population, PFS2 favored the abemaciclib arm across all subgroups of prognostic factors (Fig. 2a). The largest PFS2 benefit was also observed in patients with initial high-grade tumors (HR, 0.418; 95% CI, 0.240–0.730).

**Fig. 2:**
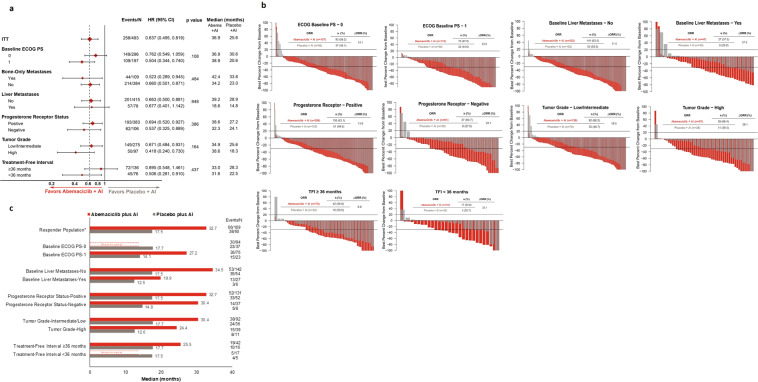
Time to second disease progression, change in tumor size, and duration of response across prognostic subgroups in MONARCH 3. **a** Time to second disease progression across ITT and prognostic subgroups. Patients who did not have a PFS2 event (second progression, or death prior to the second progression) were censored at the last available time point at which it was established that the patients did not have a PFS2 event. **b** Waterfall plots^#^ for best percentage change in tumor size across prognostic subgroups. **c** Median duration of response. ^#^ The bars for the placebo group are represented using larger width since there was 2:1 randomization. * Includes 2 responders recorded as having non-measurable disease only. Abbreviations: abema, abemaciclib; AI, aromatase inhibitor; CI, confidence interval; ECOG PS, Eastern Cooperative Oncology Group performance status; HR, hazard ratio; ITT, intent-to-treat; N, number of patients; n, number of patients in that subgroup; ORR, objective response rate; PDT, post-discontinuation therapy; PFS2; time to second disease progression or death; TFI, treatment-free interval.

In patients with measurable disease, the ORR was 62.5% (95% CI, 56.7–68.2) in the abemaciclib arm and 44.7% (95% CI, 36.2–53.2) in the placebo arm. As evidenced by the best change in tumor size and corresponding ORR, all prognostic subgroups received benefit from the addition of abemaciclib to AI, with the largest effects observed in subgroups of patients with liver metastases, PgR-negative tumors, high-grade tumors, or TFI < 36 months (Fig. 2b). Median time to response was similar between the two treatment arms (3.6 months in abemaciclib arm AI vs 3.7 in placebo arm) and generally consistent across subgroups. Responses were more durable in the abemaciclib arm (median duration of response: 32.7 months; 95% CI, 25.7–not reached) versus the placebo arm (median duration of response: 17.5 months; 95% CI, 11.6–22.2), including in poor prognostic subgroups (Fig. 2c).

No new safety signals were reported, and the safety profile was generally consistent with results disclosed previously.

This analysis of the MONARCH 3 trial builds upon a prior exploratory analysis of over 1000 patients from the MONARCH 2 and MONARCH 3 HR+, HER2− ABC trials, in which statistically significant prognostic factors were identified through a robust univariate and multivariate analysis. Following identification of the prognostic factors, the treatment effect of the addition of abemaciclib to ET was assessed in each subgroup. We present an update of the MONARCH 3 data with an additional 12 months of follow-up since the final PFS readout. This updated analysis confirmed that the addition of abemaciclib to AI statistically significantly improved PFS with an absolute PFS benefit of 13.4 months (HR, 0.525), consistent with the effect size reported at the final PFS readout (HR, 0.540)^9^. Moreover, the additional follow-up enabled this estimation of the PFS rates at 3 years, highlighting the sustained treatment benefit. The benefit was maintained across subgroups, including patients with poorer prognosis, such as presence of liver metastases, PgR-negative tumors, high tumor grade, and TFI < 36 months.

Abemaciclib plus AI also delayed the initiation of chemotherapy. Delaying the onset of chemotherapy is a meaningful endpoint for patients because of the increased toxicity often attributed to this therapy. PFS2 was statistically significantly improved across the ITT population and prognostic subgroups. While the addition of abemaciclib benefitted all subgroups, including those with a poorer prognosis, the largest PFS2 benefit was observed in patients with high tumor grade. PFS2 has also gained recognition as a critical additional endpoint in several clinical trials^10,11^. These findings are of interest in MONARCH 3 as OS data are maturing. Currently, OS has not yet been reported for any of the phase 3 studies where CDK4 & 6 inhibitors were administered as first-line treatment in HR+, HER2− ABC.

As demonstrated by the best change in tumor size and the corresponding ORR in patients with measurable disease, all prognostic subgroups received benefit from the addition of abemaciclib to AI. These results are consistent with those observed in the overall measurable disease population. Among exploratory subgroups, the largest effects continue to be observed in subgroups of patients with baseline liver metastases, PgR-negative tumors, high-grade tumors, or TFI < 36 months. Extended follow-up in the MONARCH 3 trial further confirmed the addition of abemaciclib to AI was associated with deep and durable tumor responses, including those in patients with poorer prognosis.

In previously reported subgroup analyses, additional follow-up was necessary to illuminate the treatment effect in patients with more indolent disease (i.e., bone-only disease or TFI ≥ 36 months)^8^. Now, with extended follow-up in MONARCH 3, we observed that patients with bone-only disease or TFI ≥ 36 months derived meaningful PFS benefit from the addition of abemaciclib to AI which is comparable to that of other subgroups.

Finding effective treatments for patients with poor prognostic factors constitutes a major challenge in clinical practice, as some of these prognostic factors, such as PgR-negative and high-grade tumors^12,13^, have been implicated in ET resistance. Patients with poor prognostic factors have historically experienced low survival rates, thus validating the critical need to find effective therapies for these patients^14–18^. Consistent with previously disclosed exploratory analyses, these updated subgroup analyses continue to suggest that the numerically largest benefit was observed in patients with adverse prognostic factors, such as liver metastases, PgR-negative tumors, high-grade tumors, or short TFI. Therefore, the observed impact of abemaciclib in these poor prognostic subgroups in terms of extending PFS, PFS2, and TCT, as well as improving response, is both hypothesis-generating and encouraging as we await overall survival data in this population.

## Methods

### Study design, prognostic subgroups, and assessments

MONARCH 3 (NCT02246621) was a randomized, double-blind, phase 3 trial of abemaciclib or placebo with an AI (anastrozole or letrozole, per physician’s choice) in postmenopausal women with HR+, HER2− locoregionally recurrent or metastatic breast cancer. Detailed study design and treatment were previously described^7^. Briefly, patients were randomized (2:1) to receive abemaciclib/placebo (150 mg twice-daily continuous schedule) plus NSAI (1 mg anastrozole or 2.5 mg letrozole, daily, per physician’s choice). Patients were stratified by metastatic site (visceral, bone-only, or other) and prior neoadjuvant or adjuvant ET (AI, no ET, or other). Prior systemic therapy in the advanced setting was not allowed, but patients could have received neoadjuvant or adjuvant ET if the disease-free interval was >12 months from the end of ET. Data reported here (cut-off date: 31 October 2018) come from an additional 12-months of follow-up after the final PFS readout.

The prognostic variables in MONARCH 2 and MONARCH 3 trials were identified previously^8^. Cross study subgroup analyses were evaluated for important demographic and clinical variables to define prognostic variables potentially associated with the performance of endocrine monotherapy or combination therapy. A univariate Cox model analysis, which was stratified by study and treatment arm, was used to assess each variable as potentially prognostic (independent of treatment). Variables with the likelihood ratio *p*-value < 0.05 were considered potentially prognostic. Next, the variables identified as potentially prognostic in the univariate analysis were analyzed in a multivariate Cox model analysis. Variables were selected in a stepwise fashion, with an entry *p*-value = 0.05 and a retaining *p*-value = 0.05. For the prognostic variables identified in the multivariate analysis, treatment effects for the addition of abemaciclib to ET were reported for each subgroup by study and treatment arm. Only patients with a complete record of included baseline variables were included. Patients may have more than one prognostic factor and thus may be included in multiple subgroups. The current analysis described in this report focuses solely on the MONARCH 3 trial.

The MONARCH 3 study was approved by the ethical and local institutional review boards for the sites participating in the clinical trial and was conducted according to the Declaration of Helsinki. Patients provided written informed consent before enrollment. This study was overseen by a steering committee, and safety data were evaluated quarterly by an independent data monitoring committee.

### Endpoints

The primary endpoint of the trial was investigator-assessed PFS as defined by response evaluation criteria in solid tumors (RECIST) version 1.1. Secondary endpoints included ORR (complete response [CR] + partial response [PR]), disease control rate (percentage of patients with CR, PR, or stable disease [SD]), clinical benefit rate (percentage of patients with CR, PR, or SD ≥ 6 months), duration of response (time from first evidence of CR or PR until disease progression or death), OS (time of randomized assignment until death), and safety. Exploratory endpoints of PFS2 (time from randomization to the discontinuation date of next-line [first line of PDT], or starting date of the second line of PDT or death from any cause, whichever was earlier), time to chemotherapy (time from randomization to the initiation of subsequent chemotherapy, censoring patients who died prior to initiation of chemotherapy), and time to first response (time from randomization until the first evidence of CR or PR), were also reported.

### Statistical analyses

All efficacy analyses were performed on the ITT population and exploratory subgroups. ORR was reported in patients with measurable disease. Time to response and duration of response were defined for responders only. Power calculations and methods for analyzing the primary and secondary endpoints were previously reported. Time-to-event variables were analyzed using the Kaplan–Meier method. HRs were estimated using Cox models and summarized using forest plots. Stratified HRs were reported for all ITT analyses, while unstratified HRs were reported for subgroup analyses. Waterfall plots were used to illustrate the magnitude of change in tumor size for each subgroup. Response rates and duration of response for bone-only disease subgroup are not reported as the majority of lesions were not measurable. The statistical analyses were performed using SAS (version 9.2 or later; SAS Institute, Cary, NC).

### Reporting summary

Further information on research design is available in the Nature Research Reporting Summary linked to this article.

## Supplementary information

Supplementary Information

Reporting Summary

## Data Availability

The data generated and analyzed during this study are described in the following data record: 10.6084/m9.figshare.14579409^19^. All data are contained in SAS files with the following names: adsl, adtte, adtte2, adtr, adrs. These files are housed on institutional storage and are not openly available in order to protect patient privacy. However, Eli Lilly and Company provides access to all individual participant data collected during the trial, after anonymization, with the exception of pharmacokinetic or genetic data. Access is provided after a proposal has been approved by an independent review committee identified for this purpose and after receipt of a signed data sharing agreement. For details on submitting a request, see the instructions provided at www.vivli.org: https://vivli.org/about/data-request-review-process/.

## Code availability

All statistical analyses were conducted using SAS v9.2 or later.
