# Supplementary material for: Abemaciclib as initial therapy for advanced breast cancer: MONARCH 3 updated results in prognostic subgroups
Source: NPJ Breast Cancer. 2021 Jun 22;7:80. doi: 10.1038/s41523-021-00289-7 (PMC8219718; doi:10.1038/s41523-021-00289-7)
Supplement: Supplementary file 1 — Supplementary Information [file 41523_2021_289_MOESM1_ESM.pdf]

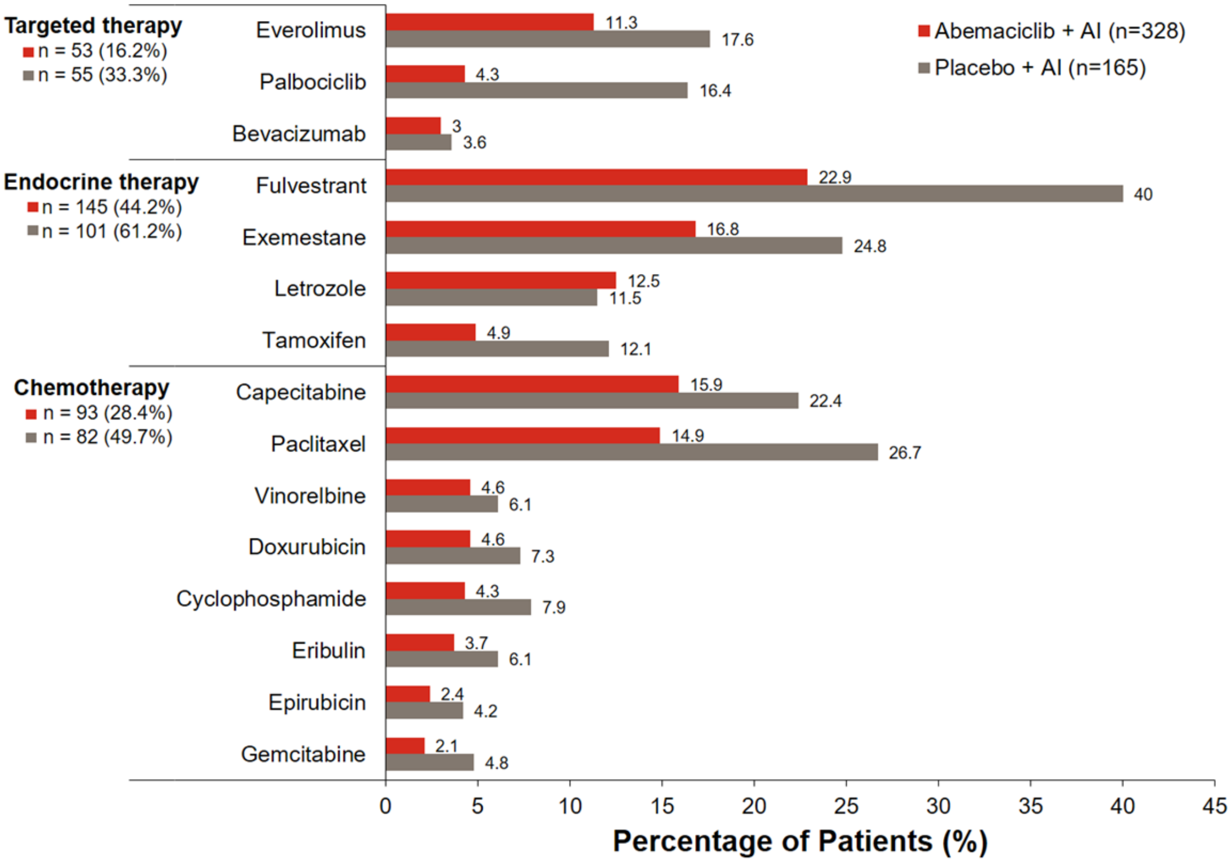

Supplementary Figure 1. Most common overall systemic therapies ≥3% of patients[1] (ITT population)

[1]All lines of post-discontinuation systemic therapy are included. Patients can be included in more than one category.

Abbreviations: AI, aromatase inhibitor; n, number of patients; ITT, intent-to-treat.
