# Supplementary material for: Abemaciclib as initial therapy for advanced breast cancer: MONARCH 3 updated results in prognostic subgroups
Source: NPJ Breast Cancer. 2021 Jun 22;7:80. doi: 10.1038/s41523-021-00289-7 (PMC8219718; doi:10.1038/s41523-021-00289-7)
Supplement: Supplementary file 2 — Reporting Summary [file 41523_2021_289_MOESM2_ESM.pdf]

nature research

Corresponding author(s): DBPR NPJBCANCER-00934R

Last updated by author(s): Jan 7, 2020

Reporting Summary

Nature Research wishes to improve the reproducibility of the work that we publish. This form provides structure for consistency and transparency in reporting. For further information on Nature Research policies, see our Editorial Policies and the Editorial Policy Checklist.

Statistics

For all statistical analyses, confirm that the following items are present in the figure legend, table legend, main text, or Methods section.

n/a	Confirmed	
☐	☒	The exact sample size (*n*) for each experimental group/condition, given as a discrete number and unit of measurement
☐	☒	A statement on whether measurements were taken from distinct samples or whether the same sample was measured repeatedly
☐	☒	The statistical test(s) used AND whether they are one- or two-sided<br>*Only common tests should be described solely by name; describe more complex techniques in the Methods section.*
☒	☐	A description of all covariates tested
☐	☒	A description of any assumptions or corrections, such as tests of normality and adjustment for multiple comparisons
☐	☒	A full description of the statistical parameters including central tendency (e.g. means) or other basic estimates (e.g. regression coefficient) AND variation (e.g. standard deviation) or associated estimates of uncertainty (e.g. confidence intervals)
☐	☒	For null hypothesis testing, the test statistic (e.g. *F*, *t*, *r*) with confidence intervals, effect sizes, degrees of freedom and *P* value noted<br>*Give P values as exact values whenever suitable.*
☒	☐	For Bayesian analysis, information on the choice of priors and Markov chain Monte Carlo settings
☒	☐	For hierarchical and complex designs, identification of the appropriate level for tests and full reporting of outcomes
☐	☒	Estimates of effect sizes (e.g. Cohen's *d*, Pearson's *r*), indicating how they were calculated

Our web collection on statistics for biologists contains articles on many of the points above.

Software and code

Policy information about availability of computer code

Data collection	No software was used
Data analysis	The statistical analyses were performed using SAS (version 9.2 or later; SAS Institute, Cary, NC).

For manuscripts utilizing custom algorithms or software that are central to the research but not yet described in published literature, software must be made available to editors and reviewers. We strongly encourage code deposition in a community repository (e.g. GitHub). See the Nature Research guidelines for submitting code & software for further information.

Data

Policy information about availability of data

All manuscripts must include a data availability statement. This statement should provide the following information, where applicable:

- Accession codes, unique identifiers, or web links for publicly available datasets
- A list of figures that have associated raw data
- A description of any restrictions on data availability

Eli Lilly and Company provides access to all individual participant data collected during the trial, after anonymization, with the exception of pharmacokinetic or genetic data. Data are available to request 6 months after the indication studied has been approved in the US and EU and after primary publication acceptance, whichever is later. No expiration date of data requests is currently set once data are made available. Access is provided after a proposal has been approved by an independent review committee identified for this purpose and after receipt of a signed data sharing agreement. Data and documents, including the study protocol, statistical analysis plan, clinical study report, blank or annotated case report forms, will be provided in a secure data sharing environment. For details on submitting a request, see the instructions provided at www.vivli.org.

Field-specific reporting

Please select the one below that is the best fit for your research. If you are not sure, read the appropriate sections before making your selection.

☒ Life sciences ☐ Behavioural & social sciences ☐ Ecological, evolutionary & environmental sciences

For a reference copy of the document with all sections, see nature.com/documents/nr-reporting-summary-flat.pdf

Life sciences study design

All studies must disclose on these points even when the disclosure is negative.

Sample size	493 patients were randomised in MONARCH3. Power calculations and methods for analyzing the primary and secondary endpoints were previously reported. The study was powered to 80% at one-sided a = 0.025 assuming a hazard ratio of 0.67 in favor of the abemaciclib arm, with a final analysis at 240 progression-free survival events.
Data exclusions	All efficacy analyses were performed on the ITT population and exploratory subgroups. ORR was reported in patients with measurable disease.
Replication	The primary endpoint of the trial was investigator-assessed PFS as defined by response evaluation criteria in solid tumors (RECIST) version 1.1. An independent central review was performed and consistent progression-free survival results were previously reported.
Randomization	Detailed study design and treatment were previously described. An interactive Web response system was used to randomly assign patients 2:1 to receive abemaciclib (150 mg twice daily, with or without food) or matching placebo plus a nonsteroidal AI (either 1 mg anastrozole or 2.5 mg letrozole). Randomly assigned patients were stratified by metastatic site (visceral, bone only, or other) and prior neoadjuvant or adjuvant endocrine therapy (AI, no endocrine therapy, or other).
Blinding	Detailed study design and treatment were previously described. MONARCH 3 is a phase III, randomized, double-blind trial of abemaciclib or placebo plus a nonsteroidal AI.

Reporting for specific materials, systems and methods

We require information from authors about some types of materials, experimental systems and methods used in many studies. Here, indicate whether each material, system or method listed is relevant to your study. If you are not sure if a list item applies to your research, read the appropriate section before selecting a response.

Materials & experimental systems

n/a	Involved in the study	
☒	☐	Antibodies
☒	☐	Eukaryotic cell lines
☒	☐	Palaeontology and archaeology
☒	☐	Animals and other organisms
☐	☒	Human research participants
☐	☒	Clinical data
☒	☐	Dual use research of concern

Methods

n/a	Involved in the study	
☒	☐	ChIP-seq
☒	☐	Flow cytometry
☒	☐	MRI-based neuroimaging

Human research participants

Policy information about studies involving human research participants

Population characteristics	Detailed study design and population were previously described. Eligible postmenopausal women were 18 years or older with locally tested HR-positive, HER2-negative locoregionally recurrent breast cancer not amenable to surgical resection or radiotherapy with curative intent or metastatic disease. Patients must have had measurable disease or nonmeasurable bone-only disease as defined by Response Evaluation Criteria in Solid Tumors (RECIST), version 1.1 and must not have received systemic therapy for advanced disease. Endocrine therapy in the neoadjuvant or adjuvant setting was permitted if the patient had a disease-free interval greater than 12 months from the completion of endocrine therapy.
Recruitment	Patients were required to provide informed consent before enrollment. The trial was conducted in accordance with the Declaration of Helsinki and was overseen by a steering committee.
Ethics oversight	The study was approved by the ethical and local institutional review boards for the sites participating in the clinical trial.

Note that full information on the approval of the study protocol must also be provided in the manuscript.

Clinical data

Policy information about clinical studies

All manuscripts should comply with the ICMJE guidelines for publication of clinical research and a completed CONSORT checklist must be included with all submissions.

Clinical trial registration	NCT02246621
Study protocol	Study protocol is uploaded as a supporting file along with the submission.
Data collection	An electronic data capture system was used in this trial. Case report form data were encoded and stored in a clinical trial database.
Outcomes	Outcomes for the trial were pre-specified in the protocol and statistical analysis plan. The primary endpoint of the trial was investigator-assessed PFS as defined by response evaluation criteria in solid tumors (RECIST) version 1.1. Secondary endpoints included ORR (complete response [CR] + partial response [PR]), disease control rate (percentage of patients with CR, PR, or stable disease [SD]), clinical benefit rate (percentage of patients with CR, PR, or SD ≥6 months), duration of response (time from first evidence of CR or PR until disease progression or death).
